# High metanephrines with adrenal hemorrhage: a diagnostic dilemma

**DOI:** 10.1210/jcemcr/luag282

**Published:** 2026-09-25

**Authors:** Maria Ortega Abad, Mohamed Eldib, Natalia Fretes Oviedo, Pratibha P R Rao

**Affiliations:** Department of Endocrinology, Diabetes and Metabolism, Cleveland Clinic, Cleveland, OH 44195, USA; Department of Family Medicine, St Elizabeth Youngstown Hospital, Youngstown, OH 44504, USA; Department of Endocrinology, Diabetes and Metabolism, Cleveland Clinic, Cleveland, OH 44195, USA; Department of Endocrinology, Diabetes and Metabolism, Cleveland Clinic, Cleveland, OH 44195, USA

**Keywords:** adrenal hemorrhage, pheochromocytoma, metanephrines, transarterial embolization, catecholamines

## Abstract

Adrenal hemorrhage (AH) may mimic hemorrhagic pheochromocytoma, particularly with markedly elevated plasma metanephrines. We report a 39-year-old woman who presented with acute right-sided abdominal pain, falling hemoglobin, and right AH on computed tomography (CT). Plasma metanephrine and normetanephrine were elevated above 3 times the upper limit of normal, raising concern for a hemorrhagic pheochromocytoma. Adrenal arteriography confirmed active hemorrhage and the vessel was successfully embolized. The day after embolization, metanephrines normalized, and adrenocortical function was intact. At 6 months, she remained asymptomatic, with normal dexamethasone suppression test and stable hematoma on CT. This case illustrates that markedly elevated metanephrines in the acute setting of AH may reflect physiologic stress rather than autonomous catecholamine secretion.

## Introduction

Adrenal hemorrhage (AH), potentially life-threatening condition, with prevalence of 0.11% among computed tomography (CT) imaging performed in emergency settings [1, 2]. The clinical presentation ranges from asymptomatic to acute adrenal insufficiency (AI) and hemodynamic collapse, making timely recognition essential [3]. The adrenal glands are inherently susceptible to hemorrhage due to their unique vascular architecture. Each gland receives a rich arterial supply from ∼50 to 60 small branches originating from 3 main arteries, yet venous outflow is limited to a small number of vessels, predisposing to venous congestion, high intravascular pressure, and subsequent hemorrhage, particularly under conditions of physiologic stress [2]. Unilateral AH is more common, and frequently presents without symptoms of AI or hemodynamic instability; however, exceptional cases with severe clinical deterioration requiring embolization can occur [4].

Multiple factors have been associated with an increased risk of AH, including invasive procedures, blunt abdominal trauma, coagulopathies, antiphospholipid syndrome (APS), sepsis, and underlying adrenal neoplasms [5]. In any patient presenting with AH, an underlying adrenal mass must be considered and systematically excluded. Pheochromocytoma warrants particular attention, as it represents the most commonly reported neoplasm associated with spontaneous AH, accounting for 48% of hemorrhagic adrenal masses in a comprehensive review of 133 reported cases [6].

The propensity of pheochromocytomas to become hemorrhagic is attributed to their hypervascular nature and the effects of catecholamine excess. Catecholamine-mediated venous vasoconstriction and thrombosis create a high-pressure intravascular environment, leading to venous rupture [2]. Additionally, despite the slow growth pattern and favorable prognosis of pheochromocytomas, with a reported median overall survival of 18 years, acute intratumoral hemorrhage can result in rapid tumor expansion due to increased intracapsular pressure, further exacerbating hemorrhagic progression and increasing the risk of shock [7-11].

Laboratory and imaging evaluation are the cornerstones of diagnosing AH and excluding underlying neoplasms. However, when the hemorrhage is acute and extensive, identification of an underlying adrenal mass on imaging may be obscured on the initial CT image [1]. The biochemical assessment of catecholamine excess is similarly challenging in the acute setting, as metanephrines may be elevated as a consequence of physiologic stress, rather than autonomous tumor secretion. We herein present the case of a female patient with unilateral AH, serum metanephrines exceeding 3 times the upper limit of normal (ULN), and hemodynamic instability.

## Case presentation

A 39-year-old woman with a history of asthma and iron deficiency anemia presented to the emergency department with sudden-onset right-sided abdominal pain, which began approximately 1 hour prior to arrival while she was working at a computer. The pain was described as sharp and stabbing and was associated with nausea and vomiting. Her last menstrual period was 9 days prior to presentation. She denied urinary symptoms, vaginal complaints, recent trauma, anticoagulant or nonsteroidal anti-inflammatory drug use, or prior history of adrenal disease and malignancy. She also denied episodic headaches, palpitations, diaphoresis, or hypertension suggestive of catecholamine excess.

Her home medication included ferrous sulfate 325 mg daily. She denied tobacco, alcohol, or recreational drug use.

On presentation, her blood pressure was 132/76 mm Hg and heart rate was 114 beats per minute. She weighed 54.4 kg with a height of 157.5 cm (body mass index 21.9 kg/m^2^). Physical examination revealed right-sided abdominal tenderness without guarding or rebound. The remainder of the physical examination was unremarkable.

## Diagnostic assessment

Initial laboratory evaluation demonstrated hemoglobin 9.8 g/dL (98 g/L), which decreased to 8.1 g/dL (81 g/L) later the same day (reference range, 11.5-15.5 g/dL; 115-155 g/L), consistent with acute blood loss, and leukocytosis with a white blood cell count of 20.3 × 10^3^/µL (20.3 × 10^9^/L) (reference range, 3.7-11.0 × 10^3^/µL; 3.7-11.0 × 10^9^/L). Two peripheral blood cultures demonstrated no growth in 5 days.

**Figure 1:**
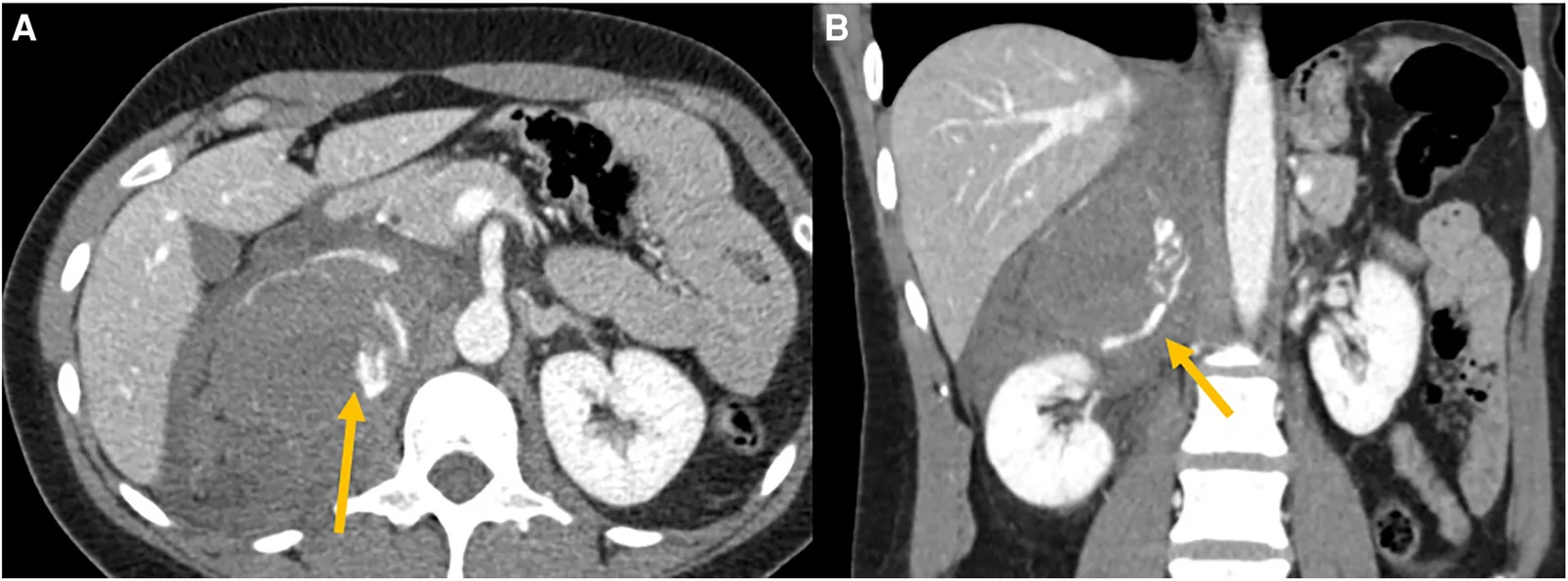
Contrast-enhanced computed tomography (CT) demonstrating a large right adrenal hemorrhage (AH). (A) Axial image showing a 5.4 × 6.9 cm right AH with acute blood products (arrow). (B) Coronal image demonstrating acute blood products on the adrenal bed (arrow).

CT of the abdomen with intravenous contrast revealed a 5.4 × 6.9 cm hemorrhage centered in the region of the right adrenal gland with evidence of active hemorrhage and a normal left adrenal gland (Fig. 1). Prior chest and abdominal CT from 2016, done to rule out pulmonary embolism, demonstrated normal adrenal glands.

Biochemical evaluation demonstrated elevated plasma metanephrine of 227 pg/mL (1151 pmol/L) (reference range, 12-67 pg/mL; 61-340 pmol/L) and plasma normetanephrine of 2380 pg/mL (12 995 pmol/L) (reference range, 18-101 pg/mL; 98-551 pmol/L), raising concern for a hemorrhagic pheochromocytoma.

**Figure 2:**
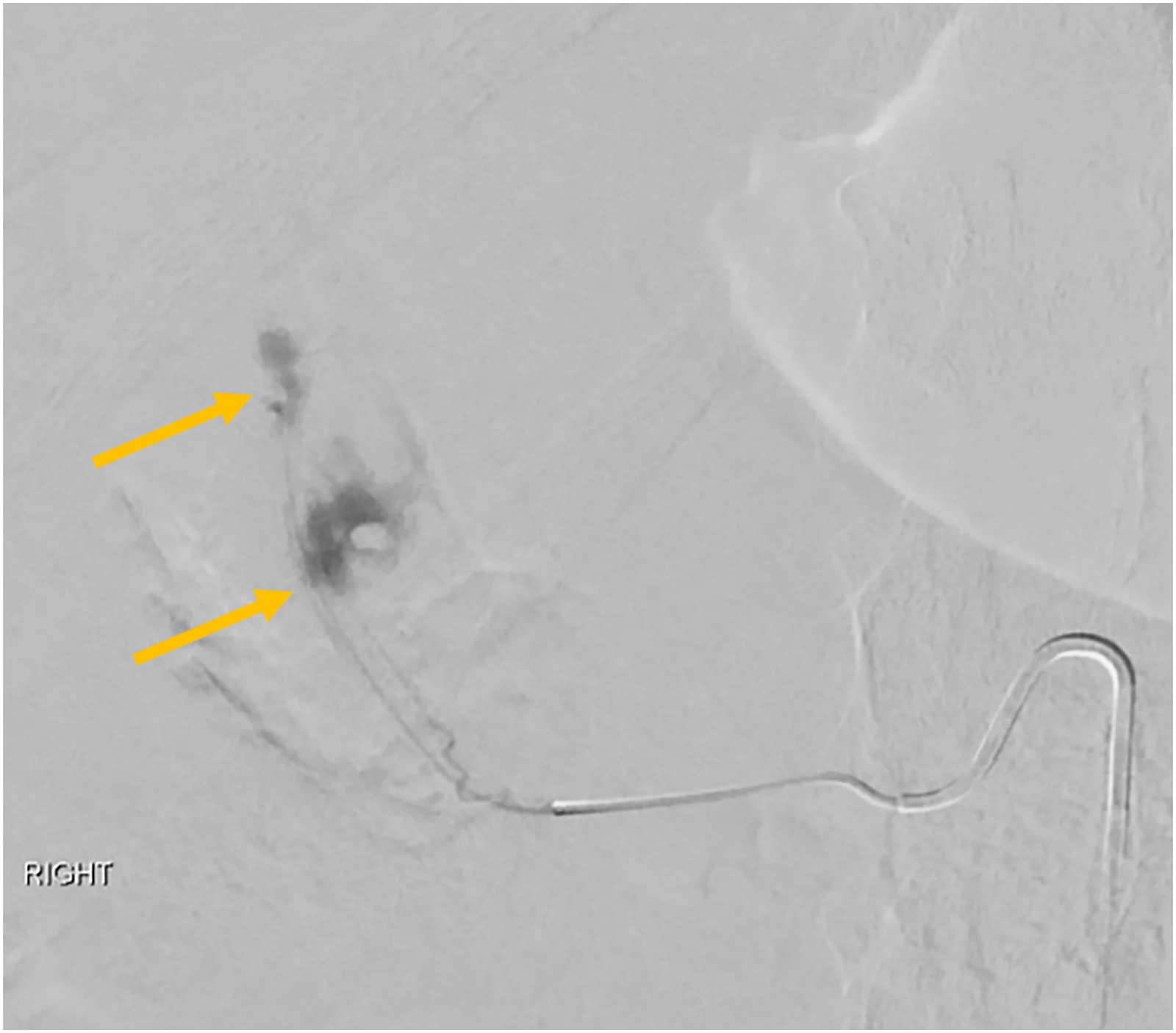
Selective right adrenal angiography demonstrating active contrast extravasation (arrows) from a branch of the right adrenal artery, consistent with ongoing adrenal hemorrhage prior to embolization.

Adrenal arteriography confirmed active hemorrhage from a branch of the right adrenal artery (Fig. 2).

## Treatment

The patient underwent embolization of the hemorrhagic vessel successfully. She remained hemodynamically stable throughout the procedure. Supportive care included intravenous fluids and pain management. No blood transfusion was required, and she did not develop hypotension or signs of AI during hospitalization.

## Outcome and follow-up

Laboratory evaluation performed the day after embolization showed normalized plasma metanephrine (81 pg/mL; 411 pmol/L) and plasma normetanephrine (256 pg/mL; 1398 pmol/L). Five days after the procedure, serum adrenocorticotropic hormone (ACTH), cortisol, and dehydroepiandrosterone (DHEA) were normal, excluding AI. Plasma renin, and aldosterone levels, as well as sodium, potassium, and chloride, were within normal limits.

A follow-up abdominal CT 1 month later demonstrated a stable right adrenal hematoma, with adjacent embolization coils and decreased surrounding fat stranding. The left adrenal gland appeared normal, without atrophy, nodules, or masses.

At 6-month follow-up, the patient remained asymptomatic. A dexamethasone suppression test at that time demonstrated appropriate cortisol suppression (post-test cortisol 0.7 µg/dL; 19 nmol/L). Her hemoglobin improved to 11.0 g/dL (110 g/L), and she was referred for follow-up adrenal CT to assess hematoma resolution and to hematology for APS evaluation.

## Discussion

Our case highlights the unique clinical challenges of spontaneous unilateral AH when hemodynamic instability coexists with markedly elevated plasma metanephrines, raising concern for an underlying pheochromocytoma. Rather than relying on a single biochemical result, clinicians should integrate the overall clinical context, biochemical evolution, and imaging findings to establish the correct diagnosis and guide management. Plasma metanephrines are catecholamine metabolites measured in blood to screen for pheochromocytoma. Normetanephrine is the metabolite of norepinephrine and is produced both within the adrenal medulla and throughout the sympathetic nervous system, whereas metanephrine, the metabolite of epinephrine, is produced almost exclusively within the adrenal medulla. This distinction reflects the tissue-specific distribution of phenylethanolamine N-methyltransferase, the enzyme that converts norepinephrine to epinephrine, whose expression is largely restricted to adrenal medullary chromaffin cells. Consequently, metanephrine production is minimal to absent in extra-adrenal sympathetic and paraganglionic tissue, and elevated metanephrine is therefore more specifically associated with adrenal pheochromocytomas [7, 12, 13].

While the diagnostic performance of serum metanephrines is accurate, with reported sensitivity of 97% and specificity of 93% for pheochromocytoma, its accuracy may be reduced in acutely ill or physiologically stressed patients [14]. Eisenhofer et al demonstrated that various conditions associated with sympathoadrenal activation can produce elevations in plasma metanephrines [8]. For instance, 30% of AH cases without pheochromocytoma had elevated serum metanephrines and/or normetanephrines, with levels within twice the ULN [2]. Current guidelines state that metanephrine levels above 3 times the ULN must not be considered false positive elevations and warrant evaluation [15, 16]. In our case, although hemodynamic instability provoked sympathetic activation and catecholamine release, metanephrine and normetanephrine levels exceeding 3 times the ULN raised concerns for pheochromocytoma.

Several strategies can aid in distinguishing true pheochromocytoma in the setting of acute AH. First, the clinical trajectory of metanephrine levels. In stress-related elevations, levels are expected to normalize following resolution of the acute illness, whereas persistently elevated levels suggest autonomous catecholamine secretion [8, 17]. Repeat biochemical testing in the outpatient setting provides more reliable results [15].

Second, follow-up imaging is essential to exclude an underlying adrenal mass. In the acute setting of a large hematoma, small adrenal lesions may be obscured on initial imaging but can become evident as the hematoma resolves. Approximately two-thirds of adrenal hematomas resolve within 15 months, with a mean reduction of 7-9 mm every 6 months [4]. Accordingly, serial unenhanced CT imaging every 3 months after the acute event is recommended until hematoma resolution or identification of an underlying lesion [18]. Contrast-enhanced magnetic resonance imaging with subtraction sequences is an alternative with higher specificity, as it removes the intrinsic hyperintensity of methemoglobin [2] Third, careful clinical history is critical to assess for symptoms of catecholamine excess (paroxysmal hypertension, palpitations, diaphoresis, and headache), which increases the pretest probability of pheochromocytoma. In our case, the absence of symptoms, the downward trend in metanephrines, and the absence of a mass on prior imaging supported the exclusion of pheochromocytoma.

Spontaneous unilateral AH is typically managed conservatively, with ∼70% of cases not requiring intervention. In a large single-center series of 363 patients, most unilateral hemorrhages resolved with observation alone, and no deaths were attributable to AH [4]. Management of hemorrhagic pheochromocytoma is guided by hemodynamic status. Stable patients should undergo biochemical testing and preoperative α-blockade before elective adrenalectomy. However, in unstable patients, urgent embolization, when available, or adrenalectomy should not be delayed pending plasma metanephrine results, which are typically available after ∼3 days [19].

In our case, the severity of ongoing hemorrhage prompted transarterial embolization to achieve definitive control. This minimally invasive approach is preferred over adrenalectomy for severe ongoing AH, as it represents a safer option. Yet, it requires identification of adrenal vascular anatomy that may represent a challenge [20]. The goal of embolization is to identify and occlude the culprit vessel, which requires active extravasation to be present at the time of angiography; the small caliber of the adrenal arteries (often <2 mm) and their variable origin from multiple sources represent a challenge requiring a skilled radiologist to perform the procedure [20, 21]. In addition, patients may develop collateral vessels and rebleed, hence, embolization does not preclude future hemorrhage, and adrenalectomy may be appropriate if embolization fails to control the hemorrhage [20].

Unilateral AH does not cause AI. A single functioning contralateral gland is sufficient to maintain the hypothalamic–pituitary–adrenal axis [4]. However, when the contralateral gland harbors pre-existing disease, such as autoimmune adrenalitis, metastatic or granulomatous infiltration, or prior surgical resection, AI can manifest. Given the high mortality risk associated with unrecognized AI, intravenous hydrocortisone (100-200 mg bolus followed by 200 mg over 24 hours) should be initiated in clinically unstable patients with suspected AH, even before CT confirmation [2]. In our case, given the patient's hemodynamic stability and normal contralateral gland, glucocorticoids were not administered.

After discharge, outpatient follow-up should focus on identifying predisposing risk factors, and monitoring for adrenal masses with CT imaging. Because unilateral AH rarely causes AI unless the contralateral gland is impaired or removed, routine repeated cortisol testing is not required. Adrenal function should be assessed if symptoms suggest AI and, when suspected, confirmed with an 8 Am cortisol level, DHEA and ACTH. APS should be excluded in patients without identifiable precipitating factors or when clinical history raises concern [2]. In our case, we believe this may have been the underlying cause of the hemorrhage; therefore, we referred the patient to hematology for further evaluation.

## Learning points

- AH can mimic pheochromocytoma, including marked elevations in metanephrines.
- Diagnosis should rely on the overall clinical context, serial biochemical testing, and follow-up imaging rather than a single measurement.
- Follow-up imaging is essential to assess hemorrhage evolution and exclude an underlying adrenal mass.
- Normalization of metanephrines after resolution of AH, together with follow-up imaging showing no adrenal mass, supports a reactive biochemical response rather than a tumoral origin.

## Data Availability

Data sharing is not applicable to this article as no datasets were generated or analyzed for this case report.
